# An Information Ontology for the Process Algebra Model of Non-Relativistic Quantum Mechanics

**DOI:** 10.3390/e22020136

**Published:** 2020-01-23

**Authors:** William Sulis

**Affiliations:** Department of Psychiatry and Behavioural Neuroscience, McMaster University, Hamilton, ON L8N 3K7, Canada; sulisw@mcmaster.ca

**Keywords:** information, process, process algebra, causal tapestry, non-relativistic quantum mechanics

## Abstract

The process algebra model has been suggested as an alternative mathematical framework for non-relativistic quantum mechanics (NRQM). It appears to reproduce the wave functions of non-relativistic quantum mechanics to a high degree of accuracy. It posits a fundamental level of finite, discrete events upon which the usual entities of NRQM supervene. It has been suggested that the process algebra model provides a true completion of NRQM, free of divergences and paradoxes, with causally local information propagation, contextuality, and realism. Arguments in support of these claims have been mathematical. Missing has been an ontology of this fundamental level from which the formalism naturally emerges. In this paper, it is argued that information and information flow provides this ontology. Higher level constructs such as energy, momentum, mass, spacetime, are all emergent from this fundamental level.

## 1. Introduction

The process algebra model of non-relativistic quantum mechanics (NRQM) was developed as an alternative foundation to NRQM which would be consistent with known results yet be free of the philosophical and conceptual conundrums that have dogged it since its conception [1,2]. The goal is to provide conceptual clarity, not necessarily improve on computational capability. It has been proposed as a complete theory of NRQM [3]. It is a causally local, contextual, realist theory which, under certain conditions, appears able to reproduce the wave functions of NRQM with a high degree of accuracy. Consistency with the results of NRQM is a necessary condition for any alternative foundation of NRQM and the process algebra model appears to meet this condition. It further claims to do so without the usual paradoxes and conceptual obfuscations [1,2]. The model proposes a discrete ontology of fundamental entities upon which the usual quantum entities supervene. Being discrete, it is divergence free. Some tentative extensions to the relativistic regime, quantum electrodynamics, have also been carried out [4,5] although much more work needs to be done to determine whether or not it can be successfully extended to include quantum field theory and the standard model, and whether it can provide movement forward on problems like the mass hierarchy, dark energy, dark matter and physics beyond the standard model. An outline of the model is presented in the Appendix A, Appendix B and Appendix C, but for technical details one should examine the original literature [1,3]. Previous arguments in support of the Process Algebra model have been primarily mathematical. The goal of this paper is to present a ontology for the model which is more physical.

Following the seminal work of von Neumann, NRQM has been formulated using the language of self adjoint operators on Hilbert spaces [6]. The process algebra model utilizes the fact that the Hilbert space of NRQM is a reproducing kernel Hilbert space [7]. Given a reproducing kernel Hilbert space *H*(*X*) with base space *X*, there exists a discrete subspace *Y* of *X* (sampling subspace), and a Hilbert space *H*(*Y*) on *Y*, such that each function in *H*(*Y*) can be lifted to a function in *H*(*X*) via an interpolation procedure. Interpolation means that if *Ψ(z)* is a function in *H*(*X*), then for each *y* ∈ *Y* there exists an interpolation function *Ψ_y_*(*z*) on H(*X*) such that *Ψ*(z) = Σ*_y_*_∈ *Y*_ *Ψ*(*y*)*Ψ_y_*(z). In fact, there are usually an infinite number of these sampling subspaces. If th subspace *Y* has the form of a regular lattice the interpolation functions may be taken to be sinc functions [7]. If the subspace has an irregular structure with sufficient density, Fechtinger-Gröchenik interpolation theory may be used instead [7]. Interpolation does not reproduce all functions on *H*(*X*) but rather a more limited set of band-limited functions, that is, functions whose Fourier transform is limited to a bounded set. This ensures the existence of a natural ultraviolet cutoff.

The process algebra model turns the above relationship around. It assumes that the discrete subsets *Y* are fundamental and generated by processes, *P*, and the value *Ψ*(*y*) assigned to a point *y* in *Y* is also generated by *P* through a sharing of specific information from prior subsets brought forward by means of some propagator *K*. In the process algebra model, the resulting wave function *Ψ*(*z*) = Σ*_y_*_∈ *Y*_ *Ψ*(*y*)*Ψ_y_* (*z*) is emergent, or derived. In keeping with the process algebra terminology, these discrete subsets will be called causal tapestries and their individual points will be called informons.

All of the necessary physics can be defined on the causal tapestry *Y* and interpolation may be used to recover all of the physics on the larger space *X*. One can then think of the discrete causal tapestry *Y* as the “real” space, and the continuous space *X* as an emergent (or even illusorsy) version. It is important to understand that the “reality” in the process algebra model consists of the causal tapestries and their associated dynamics and physics. The interpolation provides an “interpretation” of the causal tapestry which links it to the usual continuous setting of NRQM. This can be thought of as a coarse graining by a macroscopic observer. The interpolation provides a necessary check to ensure that the physics on the causal tapestry is giving rise to the correct macroscopic physics.

Suppose that the NRQM system is in a superposition of eigenstates {*Ψ_n_*} for some observable, so that *Ψ*(*z*) = Σ*_n_* w*_n_Ψ_n_*(*z*). Each eigenstate wave function *Ψ_n_* can be associated with a unique sampling space ***Y****_n_*, such that ***Y****_n_* ∩***Y****_m_* = ø for *n* ≠ *m*(for example in the case of regular lattices, add a small perturbation to the original lattice along one spatial direction so as to create a unique lattice for each ***Y****_n._).* The sampling space for the original wave function may be taken to be *Y* = ∪*_n_* ***Y****_n._* This may result in oversampling of the original wave function but that will still result in an effective interpolation [7]. Thus, a distinct causal tapestry *Y_n_* may be associated with each process ***P****_n_* generating the eigenstate *Ψ_n_* [1,2,3,4,5]. In this way every point in the subspace *Y* is associated with one, and only one eigenstate. Information propagates only between informons associated with the same eigenstate. Thus, superpositions in the process algebra model occur at the level of the wave function, not at the level of the sampling points, so that there is never any ontological confusion.

Moreover, the probability associated with the wave function of NRQM via the Born rule is likely also an emergent feature, arising from interactions among processes [1,3] (see Appendix A, Appendix B and Appendix C). The Process Algebra model appears to be a true completion of NRQM, possessing a form of (causally) local realism, all the while behaving more or less exactly as does NRQM [3]. The arguments are mathematically sound, but to make the model physically plausible, an ontology is needed, to which we turn to complex systems theory for inspiration.

The use of interpolation theory in the process algebra approach was inspired by the work of Kempf [8]. Interpolation theory has been used as a computational tool for calculations [9] but its use in providing an ontological basis for quantum mechanics appears to be relatively recent (although the search for discrete ontologies per se is quite old [10]). The work of ‘t Hooft [11] and Elze [12] on cellular automata-based ontologies is complementary to the process algebra approach. The Process Algebra per se is based on the algebraic structure of combinatorial games and provides a very general framework and language for describing generative dynamical systems. Although not yet carried out, it should in principle be possible to formulate the cellular automata approach within the process algebra. The specific process algebra model described here is not a typical cellular automaton model. First of all, the dynamics is non-deterministic (in the sense of computation theory) but may be deterministic or stochastic. The use of a spacetime lattice is a heuristic convenience but is not essential to the model. In the more general model, the spacetime structure may vary from one generation to the next, as can the spatial distribution of information. ‘t Hooft and Elze base their dynamics on discrete versions of Hamiltonian operators [11,12]. The process algebra model bases its dynamics on the propagation of information via a propagator and the operator formalism arises as an effective model in the asymptotic limit of infinite information and infinitesimal spatiotemporal scales [1,3].

## 2. Physics from the Top Up

During the past century, physicists have learned that the fundamental constituents of matter possess three characteristics that clearly separate them from the classical Newtonian ontology. First of all, these fundamental constituents are transient. Some are transient by virtue of having a short half life, so that they readily decay into other fundamental constituents. Although stable, others are transient by virtue of being able to transform into other fundamental constituents through interactions. The fundamental constituents in the Newtonian ontology were, however, considered to be eternal. Second, these fundamental constituents are subject to contextuality. It appears to be impossible to assign to a fundamental constituent a definite set of properties associated with a set of non-commuting measurements. Each ordering of successive measurements serves as a distinct context, and the measurement depends upon the context. In the Newtonian ontology such order effects are absent, often referred to as observer independence, so that properties can be attributed to the fundamental constituent. Third, some if not all, of the fundamental constituents are emergent. For example, hadrons are formed as complexes of interacting quarks, acquiring properties distinct from those of its constituent quarks. Most obviously, quarks are never free, whereas hadrons can frequently be found free. Even a basic phenomenon, a bubble chamber track, was shown decades ago to be an emergent phenomenon [13]. As noted by Anderson [14], emergence abounds and there have been a few attempts at an emergent theory of fundamental phenomena [15,16,17].

The famed biophysicist Robert Rosen expressed the opinion that physicists had much to learn from the study of complex adaptive systems, in particular living organisms [18]. They too possess all three attributes described above: they are transient, they are contextual, and they are emergent. It is possible that insight into fundamental physics can be gained by examining which aspects of complex adaptive system dynamics might be fruitfully brought done to the level of fundamental constituents. This does not imply a return to vitalism. Transience, contextuality, and emergence can be framed in entirely formal terms and applied to a wide range of dynamical systems: physical, biological, mathematical, psychological, economic or computational.

A metaphysics already exists within which to address many of the problems associated with complex adaptive systems. That is process theory, as conceived by Whitehead early in the Twentieth century [19]. Whitehead described his metaphysics as a philosophy of organism. Transience, contextuality, and emergence are fundamental to his metaphysics. He postulated the existence of a fundamental level of entities which he called actual occasions, which come into being in successive generations through the activity of processes, persist long enough so that the information embodied within them becomes incorporated into the next generation of actual occasions through something called prehension, and then fade from existence. Becoming is prior to being in his metaphysics. The actions of a process are contextual. Fundamental constituents are emergent, and supervene upon actual occasions.

Whitehead proposed that reality was generated by process. It does not exist in its entirety in some eternal form but rather is generated moment to moment, a version of presentism [20]. Since the advent of relativity, modern physicists have denied the existence of a transient now [21,22] although in recent years others have stepped up to suggest otherwise [23,24,25]. The main argument against a transient now is the absence of a notion of simultaneity within the relativistic framework. However, as pointed out by Wigner [26], what special relativity actually asserts is the non-existence of any universal frame of reference. A global frame of reference is a mathematical fiction. No observer can ever directly observe any events that are space-like separated from them. They can, however, reconstruct a surface of simultaneity as they move forward in time by keeping track as information about previously simultaneous events arrives. The reconstructed surface of simultaneity demonstrates that the surface of simultaneity once existed even though it was not directly observable. An observer can only ever experience their own past light cone. One cannot speak of how the universe is, only how the universe was. One can no more prove that the universe is eternal and of block form than that it consists of a transient now.

## 3. The Role of Continuity

In Whitehead’s process theory, actual occasions are distinct, discrete wholes. The Newtonian ontology treats spacetime as a continuum, infinitely divisible, and described mathematically by continuous structures. Research into quantum gravity has strongly supported the idea that the continuous appearance of spacetime breaks down as one approaches Planck length and time. This appears to be necessitated by the Heisenberg Uncertainty Principle, which places limits on the accuracy with which measurements may be carried out at the smallest scales. In the context of quantum non-locality, Gisin [27,28] and colleagues studied the situation of 4 quantum observers and constraints on correlations among their respective measurements. They were able to find an inequality involving various correlated measurements, assuming the principle of continuity and a constant, finite but unspecified superluminal speed v (c < v < ∞) of propagation of any hidden signals, as well as a quantum state which violated the inequality. Gisin [27] concludes that either the principle of continuity must be violated, or superluminal signalling must be possible. He appears to be more in favour of abandoning the principle of continuity although comments that both options are problematic. He writes “Note that the finding of such a speed would falsify both quantum theory and relativity, a result not many physicists are willing to envisage” [27] (p. 10). Bancal et al. write “This gives further weight to the idea that quantum correlations somehow arise from outside spacetime, in the sense that no story in space and time can describe how they occur” [28], (p. 4). The idea of process in the Process Algebra model provides just such a trans-spatiotemporal entity capable of generating spacetime events.

Interpolation, as pointed out by Kempf [8], can provide a bridge between the discrete and the continuous. He showed that it is perfectly possible to have a discrete reality at the fundamental level and the appearance of a continuous reality at the macrolevel. Even if reality only manifests at discrete spacetime points, it is possible to calculate the values at intermediate spacetime points through the interpolation procedure. In essence, the appearance of continuity is illusory and is a result of a fundamental inability to resolve spacetime at any smaller scale. The illusion of continuity is widespread. Organisms, matter, motion pictures, all appear continuous at the macrolevel, but this breaks down at smaller scales. A discrete reality at the lowest level eliminates the problem of divergence which plagues so many continuous models. Heuristically, one can switch easily from the discrete to the continuous through the interpolation procedure. Both models will agree in value at the sampling points. The integral of the continuous function over the base space X equals the sum over the sampling set Y, so computation is often unaffected.

This provides a step towards resolving the problem of wave-particle duality. If the temporal scale is that of Planck time, *p_T_* and the spatial scale that of Planck length *p_L_*, then individual sampling points become unobservable to a macroscopic observer. Heisenberg uncertainty will prevent the resolution of events to that level. A macroscopic observer will only appreciate the interpolated, continuous wave function. According to the process algebra model, processes will interact based upon the appearance of individual informons, but the actions of these processes are not directly observable. In the two-slit experiment, the overall process *P* can be written as a sum of right and left subprocesses, *P_L_* and *P_R_*. The issue is not particle or wave; the issue is whether or not information is allowed to flow between the subprocesses during the generation of the informons. When information is allowed to flow, as when interference is permitted, *P* = *P_L_* + ^ *P_R_*, using the process algebra free sum. When information is not permitted to flow, as when interference is blocked, *P* = *P_L_* + *P_R_*, the Process Algebra exclusive sum. The processes in the two cases are *not* the same as would be implied in the NRQM formalism. In both cases, measurements are triggered by the appearance of individual informons, but the coupling between the particle and the measurement apparatus will depend upon the local process strength, which in turn depends upon how information was allowed to flow during the generation of the informons. In both cases, the processes will generate a discrete causal tapestry which in particle-like but unobservable and an emergent wave function which is wave-like at the macroscopic level.

The suggestion that the fundamental constituents of reality be discrete and reside within a discrete spacetime thus appears reasonable and consistent with known physics.

According to Whitehead, actual occasions are generated by processes, successively, one complete generation following another. This imparts discreteness to time. But what time? Since processes generate spacetime, it is possible that the action of process takes place in a time outside that of our usual 4-dimensional spacetime. The idea of a two-time physics is not new [29,30] but has not been widely accepted. In such a case, the action of process would attribute to each actual occasion a location in a 3 + 1-dimensional spacetime corresponding to observable reality such that each complete generation forms a discrete sampling of a space-like hypersurface, that is, they appear to occur simultaneously. The process would therefore generate these informons in the 2nd time but they would exist only in the normal 3 + 1-dimensional spacetime. This is similar to the role that a second time plays in stochastic quantization where a stochastic process relaxes in the 2nd time to give rise to phenomena registered in our normal time [30]. Another possibility is that the action of process occurs according to proper time. This has the advantage that it would be invariant under relativistic transformations. In this case the actual occasions could be assigned different time coordinates while still forming a discrete sampling of a spacelike hypersurface. However, if the proper time required for the creation of a complete generation of actual occasions was sufficiently small, say a small multiple of Planck time, they could be treated as if they occurred simultaneously, without much loss of accuracy. It is possible that a universal proper time might exist for all processes, otherwise a multi-time structure such as that proposed by ‘t Hooft [11] might be required.

## 4. The Ultimate Determinant of Events: Energy Versus Information

Classically one of the fundamental concerns of physics was the study of motion. Physicists studied the laws governing how physical objects move through spacetime. Physical objects, being inanimate, move in a reactive manner. They do not act or behave. They are passive, merely responding to the vicissitudes of energy as it is distributed in spacetime, always following paths which extremize some function or another according to the principle of least action. In the standard block universe model of reality, nothing really happens. All events exist simultaneously. Each physical object is associated with a set of spacetime locations which form a history, and physicists study how these different histories relate to one another. Motion is an illusion, a by-product of histories being more than single spacetime points.

The process theory view is strikingly different. Spacetime is no longer a pre-existing, eternal entity. Instead, spacetime, like the physical entities manifesting within it, is generated by processes. Prior to the appearance of an actual occasion, a spacetime point is merely a conceptual potentiality. It is only realized once an actual occasion is associated to it and it becomes identified with *something*, perhaps a Bell beable [31] with which a measurement apparatus can interact. In the Process Algebra model, spacetime consists of the collections of actual occasions that are generated by processes. Each actual occasion both represents and manifests an element of spacetime. Since the assignment of a spacetime point to an informon is a matter of interpretation (observation) in the Process Algebra model, there is a “fuzziness” associated with the localization of an informon. Indeed, the only fixed aspect is the causal distance between an informon and the prior informons within its content (see the Appendix A, Appendix B and Appendix C for details). Since processes, according to the process algebra model, generate spacetime, they should, logically, exist outside of that spacetime. The concept of process thus provides a candidate for the trans-spacetime determinants of reality that Gisin has referred to [27]. The exact nature of processes is as yet unspecified within the model. For the moment they are considered to be primitive constructs.

In the Process Algebra model, processes are free to generate an actual occasion and to locate it in relation to any other actual occasion in any manner possible. There are only two restrictions. The first is that an actual occasion cannot be identified or attributed to a location assigned to any other actual occasion. Second, the information of a prior actual occasion can propagate to a nascent actual occasion only causally. In keeping with special relativity, no signal can propagate at greater than light speed, and propagating information constitutes a signal which must respect this principle.

An actual occasion comes into being, persists until its information has been incorporated into the nascent actual occasions, and then fades away. The triad of prior generation, generating processes and nascent generation form the compound present [20] of the process model. Everything is either in a state of becoming or of fading away. Everything is happening, and nothing is eternal, except for the processes, and even they shift between activity and inactivity.

It is important to recognize that actual occasions do not move. They come into being and then fade away. They manifest a spacetime location; they do not move from one spacetime location to another. They do not interact with one another. Processes interact, actual occasions do not. There is no energy associated with actual occasions because there is no motion against which to define a kinetic energy and no interactions to define a potential energy.

Energy is frequently, and somewhat casually, said to underlie everything in physical reality. Energy is said to be exchanged, much like money, or property, or other material objects are exchanged. This way of thinking suggests that energy is a thing in its own right, which physical objects can possess. If this is so, then it should be possible to assign a definite quantity of energy to any physical object. However, consider the following thought experiment. Consider a solitary observer in an otherwise empty universe. They possess a rocket backpack. They have a clock with which they can time the duration of firing of the rocket. Initially the rocket is quiet. They are in an inertial frame, and so cannot determine whether or not they are in motion. Thus, their kinetic energy is 0. Their potential energy is also 0 since there are no other entities with which they could interact. Now suppose that the rocket fires for a duration *t* and produces an acceleration *a*. They need not measure it. Assume that the manufacturer told them so. During the firing of the rocket, they feel the acceleration, which according to the equivalence principle would feel like being in a gravity well. After the rocket has fired, they return to an inertial frame. They are still alone in the universe. They still cannot detect if they are in motion even though, intellectually, they should be moving with velocity *ta*. But they have no means to detect that. As far as they know, they are still at rest. They still have no kinetic energy even though to an external observer they should have kinetic energy ½ *m*(*ta*)^2^. But such an external observer does not exist, at least not in our universe.

Suppose now that there are two observers, one of rest mass *m*, the other of rest mass *n*. The two observers face each other and move towards one another along their line of sight with relative speed *v*. To the first observer, the second observer approaches with speed *v*, momentum −*n**v*** and energy ½ *nv*^2^. The total momentum therefore is −*n**v*** and energy ½ *nv*^2^. To the second observer, the first observer approaches with speed *v*, has momentum −*m**v*** and energy ½ *mv*^2^. The total momentum is therefore -*m**v*** and energy ½ *mv*^2^. Neither the total momentum nor the total energy is the same for both observers. Nevertheless, in any interaction between them, the total momentum and total energy will remain constant, albeit with different constant values.

Momentum and energy can only be defined with respect to a relationship between physical objects. Energy has no intrinsic value in and of itself. If energy were a natural kind, an actual something passed between physical objects, then it should be possible to assign to it a definite value. But it can be assigned any possible value in any frame of reference. All that is required is that conservation of energy holds. That is, the bookkeeping should balance out in the end, but otherwise the numbers attached to independent events can be assigned at will. Momentum is similar in that it too cannot be assigned to single physical objects but only to relations between objects.

From an ontological perspective, Noether’s theorem [32] shows that momentum and energy describe relations between dynamical trajectories of physical objects. They are a consequence of symmetries in the equations of motion that govern such trajectories. They are not actually properties of the objects per se. They have meaning only in relation to trajectories. Once assigned, a value of energy provides information about which trajectory a physical object might be following. Interactions between physical entities ‘can result in changes of trajectories, and therefore changes in the assigned energy’. This does not necessarily imply that “something” must be exchanged between the entities, but it does denote that a change in the states of the entities has taken place. That is, information regarding the trajectories of the entities has changed. An interaction between entities thus results in a change in information.

Since energy and momentum reference trajectories, it would seem more reasonable that they be associated with the generator of the trajectory, which in the Process Algebra framework, would be the generating process. This does not eliminate the observer dependency of these quantities. One possibility is to try to link these two to the causal structure of the content sets within the informons of a causal tapestry. It is possible that, as far as reality is concerned, there is a preferred frame of reference against which such quantities can be measured, but that this frame of reference is inaccessible to macroscopic observers. If so then it might be possible to recover the idea of energy as a natural kind. This idea is still in its infancy.

As physics evolved over the centuries, its focus shifted from a study of motion and forces to a study of energy. Information has been gaining attention in physics [33,34], primarily through the relationship between Shannon’s concept of information [35] and the physical concept of entropy, which mathematically are the same but for a sign. The Shannon concept of information lacks however, any reference to meaning. He wrote [35] (p. 31): “The fundamental problem of communication is that of reproducing at one point either exactly or approximately a message selected at another point. Frequently the messages have meaning, i.e., they refer to or are correlated with certain physical or conceptual entities. These semantic aspects of communication are irrelevant to the engineering problem. The significant aspect is that the actual message is one *selected from a set* of possible messages. The system must be designed to operate for each possible selection, not just the one which will actually be chosen since this is unknown at the time of design” (SIC).

In the setting of complex adaptive systems, however, energy plays a subordinate role. Most organisms are adapted physiologically to act within a wide range of energy states and flows. An organism can carry out the same behaviours whether hungry or sated, and sometimes even when starved or obese. Energy is necessary for an organism to act, but energy alone does not determine the specific behaviour which is carried out. The main determinants are physiological, psychological and ecological, and each of these influences the organism through information. The information used by organisms, unlike that studied in engineering or physics, is meaning laden, or salient, and transferred from one organism to another through either signals or signs. The term salience is preferred to meaning since it does not imply the presence of an entity which can interpret the meaning. It merely implies that the information creates a difference. The study of signs in a biological context is called biosemiotics [36]. The role of information in determining the behaviour of organisms has become prominent in recent years. In the context of fundamental constituents, Whitehead believed that information played a central role along with energy.

Information propagates from prior actual occasions to nascent actual occasions and is incorporated into the nascent actual occasions through prehension. The simplest mechanism by which information could propagate from actual occasion to actual occasion is diffusion. There have been several attempts to describe quantum mechanics as a diffusion process [37,38] so this is a reasonable initial assumption.

Information from a prior actual occasion can be incorporated into many nascent actual occasions; likewise, a nascent actual occasion may receive information from many prior actual occasions. It appears that information has an inherent tendency to flow. Whitehead’s concept of prehension is, however, elusive, so a general formal model of how information from prior informons might be incorporated into nascent informons is not yet in hand. Nevertheless, in the setting of NRQM, the essential information is contained within the wave function. There is already a formal theory describing how wave function amplitudes along different paths are to be incorporated into a final wave function. That is provided by Feynman’s idea of path integrals [39]. Amplitudes sum over space and multiply over time. A process can, in principle, be decomposed into a Process Algebra free sum of subprocesses, one for each informon in the prior causal tapestry *I*, ***P*** = Σ^*_n_*_in *I*_ ***P_n_***. This is akin to the decomposition in the two slit case but now extending it to every informon in the prior causal tapestry. Assuming that the information possesses an algebraic structure, it would be very natural if whatever information is associated with each subprocess were to be summed by the overall process when generating informons. This would provide a natural homology between summation among processes and summation among information. Likewise, when one process follows another, represented in the Process Algebra by concatenation, ***PQ***, it would be natural if the information being incorporated into the nascent informons would be a product of information prior to the actions of ***P*** and prior to the actions of ***Q***. The Feynman rule for calculating amplitudes over paths forms a natural fit with the Process Algebra formalism. While not a proof, this informally motivates the use of the propagator formalism for incorporating information in the process algebra model of NRQM.

## 5. Process Strength

The most basic kind of information that could be passed from actual occasion to actual occasion is that of local coupling effectiveness (and thus local process strength). Processes are assumed to interact with one another according to Trofimova’s compatibility principle [40]. This principle is based on observations of complex adaptive systems where interactions appear to depend upon the level of compatibility between systems. Indeed, among the elementary particles, interactions only occur between particles that can exchange certain mediating particles–photons, gluons, mesons. Incompatible particles do not interact. The local coupling effectiveness and local process strength provide a contribution to the determination of compatibility between two or more processes.

The units of the NRQM wave function are volume^−1/2^. The squared norm of the wave function has long been interpreted as a probability density according to the Born rule, while the wave function itself seems to have no interpretation. This is unsatisfying for something that is supposed to provide a fundamental description of the state of a quantum system. From the Process Algebra perspective, the wave function should better be interpreted as a measure of local compatibility, while its squared norm is interpreted as a process strength density. Instead of saying that a particle is detected at a particular location one says that the particle process and measurement apparatus process achieved compatibility and interacted at a particular location. The particle process need not be “localized” to that particular location. Indeed, the particle process will be generating informons over a spatial region. It just happens that particle and measurement apparatus informons at that location were deemed compatible and triggered the interaction that resulted in a “measurement”.

Classical information theory is about meaningless information, being concerned with the carrying capacity of signals rather than the content of those signals [35]. Semiotics on the other hand concerns itself with signs and with meaning, and how meaning is transmitted by virtue of signs [36]. In semiotics, meaning is closely related to form. Consider a key and a lock. The key is compatible with the lock if it possesses the correct length, the correct grooving to match the tumblers and is oriented in the correct direction when insertion is attempted. The form of the key is essential to its function. Likewise, in physics, the form of the information may be critical to determining the behaviour and types of interactions between physical objects. Physical form is an important feature of information shared between organisms. Information that can be readily disseminated (such as by light or sound) serves different functions than information that can only be transmitted locally via direct contact or line of sight, or through stigmergic artifacts (or books or phones in the case of humans). Physical form is also important in chemical reactions, particularly in organisms. It underlies the binding of neurotransmitters and drugs. Is it possible that the mathematical form of information is also important? Fundamental particles are associated with different kinds of mathematical representations (scalars, spinors, vectors, tensors) defined over various number fields (integral, real, complex, quaternion, octonion). This is mere conjecture, but could it be that the different mathematical forms associated with the fundamental particles serve an informational role? The spin-statistics theorem shows that the type of spin serves to determine the effect of permutations on the wave function. Does this have an informational interpretation? Knowledge of spin type appears to inform about other features of the particle. Can this be developed further?

Since compatibility involves information and meaning (whether physical or abstract) [40], it is expected that the local coupling effectiveness would possess a form which expresses this meaning. The particular mathematical form of the local coupling effectiveness should express, when formally combined with the mathematical forms of the local coupling effectiveness of candidate processes, the determination of compatibility between the processes. When viewed from a process perspective, the form of the wave function no longer appears so inscrutable. The choice of number field need no longer be tied to simplistic connections to measurements, which represent only a specific set of interactions. Instead the number field and the mathematical form represent deep algebraic relationships between the associated processes, presumably expressing some deep symmetries or relationships.

## 6. Intrinsic Versus Extrinsic Characteristics

The formal description of informons includes two parameters, a set of intrinsic properties ***p*** and a set of extrinsic properties ***Φ***. Intrinsic properties are inherited from the process which generated the informon. Technically speaking, they are not really an ontological aspect of the informon per se, but rather they serve to identify which process generated it and which process will propagate its information forward into the nascent collection of informons. These intrinsic properties distinguish one process from another. Since processes are held to exist outside of spacetime (being generators of spacetime), they must exist apart from any particular observer (since they generate the informons upon which any observer supervenes). Thus, any intrinsic property should be an invariant of the process, independent of any particular reference frame used to model the process. Charge and rest mass would be two obvious empirically observable candidates for intrinsic characteristics. The mathematical type of the wave function (scalar, spinor, vector, tensor) would seems to be another candidate intrinsic characteristic. Causal distance is another invariant which can be attached to an informon as an intrinsic characteristic (referenced to the collection of prior informons) and which can be attributed to the informon itself. Causal distance appears in the structure of the content. The causal structure suffices to define the topology of any causal manifold into which the informons are embedded [41]. The local coupling effectiveness is another invariant which is attributed to the informon itself although it has its origin in the action of the generating process.

Extrinsic characteristics are those which are observer dependent. Unlike the intrinsic characteristics, the external characteristics must be referenced to some reference frame or to some observing system and will vary from frame to frame. Position, momentum, energy are all external characteristics as are the interpolation functions which enter into the local Hilbert space interpretations. They can be freely chosen although their usefulness and effectiveness will be affected by poor choices. They are not part of the reality at the informon level but serve as bridges between the informon level and the macroscopic level of the observer. There are deep questions related to whether momentum and energy can be associated with a generating process as intrinsic characteristics and which will not be addressed here.

## 7. Conclusions: The Primacy of Information

In Whitehead’s process theory, information plays a fundamental role. However it is only meaning laden information which makes a difference in the generation of actual occasions by processes. Energy, while necessary, is no longer sufficient to determine the flow of events. This is true for complex adaptive systems and it is suggested that this is also true for fundamental physical processes as well. The process algebra model explicitly postulates that below the standard level of fundamental constituents there exists a level of actual occasions. These manifest at Planck scales, and so are effectively unobservable to macroscopic observers. Information is propagated among actual occasions by processes in a manner akin to a diffusion process, following a Schrödinger equation. The standard fundamental physical constituents manifest as emergent from causally coherent collections of actual occasions as generated by processes. Spacetime, and the entities manifest within it, are all generated by processes. The resulting reality, according to process algebra models, appears to be realist, causally local, and contextual, divergence free, paradox free, and free of conceptual confusion [1,3].

## Appendix A. Summary of the Process Algebra Model

There have been several earlier attempts to incorporate ideas of process and information into physics [42,43,44,45,46,47,48] but none have gained widespread acceptance or usage. Trofimova [49,50] has proposed several process algebra based formalisms for describing the principles of transience which govern processes in functional constructivism. Her approach to process algebra uses several functional differentiation classes, a concept of “performance” and several universal process-trends. From this perspective there is no one-one correspondence between behaviour at different spatiotemporal levels. Instead, many different processes give rise to similar sets of behaviours, so that there is no simple correspondence by means of which one can formally move up or down the hierarchy of dynamical systems of some entity.

The process algebra model, which is the focus of this paper, brings ideas from Whitehead’s process theory [19], interpolation theory [7,8], combinatorial game theory [51] and semiotics [36] together to provide a new mathematical language for describing complex systems. The Process Algebra model was developed as a reformulation of non-relativistic quantum mechanics which could serve as a proper completion [3]. Whitehead’s actual occasions are modeled as informons. Informons are generated sequentially through the actions of processes. Each informon takes the form [*n*] < ***p***, ***Φ***, ***Γ*** > {*G*} where (1) *n* is a heuristic mathematical label, (2) ***p*** is a structured set of intrinsic properties, (3) ***Φ*** is a structured set of extrinsic properties, (4) ***Γ*** is the local coupling effectiveness, and (5) *G* is a causally ordered collection of informons called the content. The idea of the content is based on a related concept of Markoupoulou [52]. The union of content sets over all informons in the causal tapestry must itself form a causal set [41].

The local process strength at an informon *n* is given as ***Γ * Γ***. The information residing in the informons of the content is utilized by the generating process to create the informon. The intrinsic properties ***p*** are attributed to the generating process ***P*** and imparted to each informon generated by ***P***. The extrinsic properties are unique to each informon but are frame dependent. There are three additional intrinsic characteristics of process. Each process, during a single action cycle (round), incorporates information from a fixed maximum number *r* of informons into each informon which it generates. Each process carries out a fixed number *N* of rounds. During a round a process may generate a single informon (primitive process) or multiple (*R*) informons (compound process). These parameters vary from process to process. In interactions the total *N* value is constant.

The collection of informons generated after *N* rounds is called a *causal tapestry.* Given a prior causal tapestry ***I***, the action of a process ***P*** is to generate a nascent causal tapestry ***I***’, following which the elements of *I* are erased. Information only flows causally from prior to nascent informons, represented by the causal ordering of informons in the content set. Information *never* flows within a causal tapestry so that there are no causal relations between the informons within a causal tapestry (thus it forms a causal antichain).

In the context of NRQM, there are two main extrinsic characteristics. First, each informon *n* is interpreted as a point *x_n_* (causal manifold interpretation or embedding) in some causal manifold ***M***. Its content set *G* causally embeds into ***M***. Each causal tapestry forms a causal antichain in ***M***, and thus represents a discrete sampling of a spacelike hypersurface in ***M***. Second, each informon *n* is associated with a local Hilbert space interpretation of the form *φ_n_* (*z*) = ***Γ****_n_ f_n_* (*z,x_n_*). Each causal tapestry is associated with a global Hilbert space interpretation over the causal manifold of the form Ψ(*z*) = Σ*_n_ φ_n_* (*z*) = Σ*_n_* ***Γ****_n_ f_n_* (*z,x_n_*). When the informons of a causal tapestry embed into the causal manifold as a discrete lattice, it is possible to replace each *f_n_* by a spatial translation (*T_xn_ f*(*z*) = *f*(*z−x_n_*)) of a single generic sinc function *g*(*σ,z*) = sin (*σz*)/*σz*, so that Ψ(*z*) = Σ*_n_* ***Γ****_n_ T_xn_ g*(*σ,z*).

Sinc interpolation requires the use of a lattice embedding into the causal manifold with lattice spacing consistent with the Beurling density [53]. Maymon and Oppenheim [54] have shown that non-uniform embeddings will still provide a highly accurate approximation using sinc interpolation so long as the spatiotemporal separation error is small. A more realistic model requires the use of non-uniform embeddings and more sophisticated interpolation techniques, such as Fechtinger-Gröchenik theory [7].

An important concept is that of epistemological equivalence. Epistemological equivalence of two processes ***P*** and ***Q*** means that the global Hilbert space interpretations *Ψ*(*z*), *Ψ*’(*z*), respectively, are equal as functions over the causal manifold. In other words,

*Ψ*(*z*) = Σ*_n_****Γ****_n_ f_n_*(*z,x_n_*) = *Ψ*′(*z*) = Σ*_m_****Γ****′*_m_ f_m_(*z,x_m_*)

The significance of epistemological equivalence is that if one deals only with epistemologically equivalent processes then the specific details of informon generation do not matter. Thus one can choose heuristic representations of processes for the purpose of developing theory, so long as those representations result in effective physical theories. A useful representation of process is as a two-player combinatorial game. Different strategies for playing the game can be examined. Strategies can be chosen for computational or theoretical convenience so long as they result in epistemologically equivalent processes. Epistemological equivalence is rather like gauge invariance as far as computation is concerned.

For example, values for *r*, *R* may be chosen to be universal for all processes, or dependent upon some intrinsic characteristic of the process, such as the norm of its energy-momentum vector. Moreover, the strategy used by the process to generate informons may be chosen to be deterministic, non-deterministic or stochastic, so long as the resulting processes are epistemologically equivalent.

In the context of NRQM, local coupling effectiveness is taken to be the value of the wave function at a given informon, and the global Hilbert space interpretation becomes the wave function for the quantum system. In the Process Algebra framework, a decomposition of a wave function as a sum of the form Ψ(*z*) = Σ*_n_ w_i_ Ψ_i_* (*z*) implies that the informons are being generated by a process ***P*** which can be decomposed into a sum of primitive processes as ***P*** = Σ*_i_ w_i_* **P***_i_*, where each subprocess *w_i_**P**_i_* generates the wave function contribution *w_i_ Ψ_i_* (z). Moreover, the set of informons associated with each subprocess can be chosen to be separate from those of any other subprocess. Thus each informon represents a contribution from one and only one physical state. There is no confusion of ontological states within the process algebra framework.

Measurement within the process algebra framework is defined as an interaction between a system process ***P*** and a specialized measurement apparatus process ***M***. The measurement apparatus process must possess additional features, such as the existence of multiple stable attractors each corresponding to a specific measurement value, which confer upon it the status of measuring something. Interactions with measurement apparatus processes are no different than with any other processes, and so are triggered by the generation of informons according to the compatibility between the processes. This compatibility Ξ(***P,M***) is a function **of** the local compatibilities, Ξ(***P,M***) = *f*(***Γ****^P^_n_* ***Γ****^M^_m_*). The probability of an interaction taking place ***Π***(**P,M**) is in turn a function of the compatibility, ***Π***(**P,M**) = *χ*(Ξ(***P,M***). The precise form of these functions depends upon the particular case. The Born rule is expected to arise from these interactions and from the compatibility, but a precise derivation is not yet in hand.

It can be shown that all of the information necessary for carrying out the actions of process and thus the wave function resides within the informons of the causal tapestries, not the causal manifold or the Hilbert space [1,3]. Thus, the physics resides solely within the causal tapestries, enabling the assertion that reality resides within the causal tapestries, while our perception of a continuous spacetime with continuous entities and events is a consequence of the interpolation procedure.

The wave function of the process algebra model is an ontological wave function, meaning that it describes a particular instance of a causal tapestry, a particular collection of generated informons, a particular state of reality. A significant difference between NRQM and the Process Algebra model appears when computation is the goal. Every action of a process potentially results in a different causal tapestry, with a different set of informons, different causal manifold embeddings, and different global Hilbert space interpretations. To take these different possibilities into account, which is necessary in order to carry out computations, one must consider the process covering map. This is a set valued map which associates to each process ***P*** and to each causal tapestry ***I*** a set of global Hilbert space interpretations {*Ψ_i_*}, one for every possible causal tapestry generated by ***P*** with ***I*** as prior tapestry. This map Π***_p_*** (***I***) = {*Ψ_i_*} is called the process covering map. It holds for single processes. A more complicated map, the configuration space covering map, holds in the case of compound processes [1,3].

It can be shown [1,3] in the asymptotic limit as Planck length, Planck time tend to 0, *r, N* → ∞, that Π**_p_** (***I***) → {*Ψ*(*z*)}, a single wave function. Thus, in the case of a primitive process, in the asymptotic limit, the process generates only a single wave function which corresponds to the usual NRQM wave function. Thus, in the case of a primitive process, the wave function becomes *both* ontological and computational. This is not true in the case of compound processes, so that the ontological wave function which describes a single instance of reality, and the computational wave function, which is used for making predictions, are no longer the same [1,3]. It is possible that the failure to appreciate the distinction between the case of a primitive process and that of a compound process resulted in the persisting confusion as to whether or not the wave function is ontological or epistemological. No such confusion holds in the Process Algebra framework.

The process covering map gives rise to a correspondence between processes and (set-valued) operators on the space of global Hilbert space interpretations. The standard operator formalism is thus an emergent feature of the process algebra model arising in the asymptotic limit of infinite information and infinitesimal scale.

## Appendix B. The Kernel Strategy

To demonstrate the application of the Process Algebra model to NRQM, let us consider the simplistic situation of a single scalar particle whose dynamics is governed by the Schrödinger equation. This is merely an illustrative example and not meant to be representative of all Process Algebra models. To simplify matters, let the causal manifold be Euclidean 4 space, R^4^ and assume further that informons are generated on a discrete regular 4-D lattice which embeds in *R*^4^, having one time dimension of minimal length *p_T_* and three spatial dimensions, each of minimal length *p_L_*. The use of a regular lattice allows the use of sinc interpolation, which is simpler.

Let *g*(*t*,*x*,*y*,*z*) = sinc (π/*p_T_*)(*t*) sinc (π/*p_L_*)*x*) sinc (π/*p_L_*)(*y*) sinc (π/*p_L_*)(*z*) be the 4-dimensional sinc function where sinc (a)(*x*) = sin (a*x*)/a*x.*

The 4-dimensional translation is defined by *T*(*a,b,c,d*)*g*(*t*,*x*,*y*,*z*) = *g*(*t−a,x−b,y−c,z−d*).

Let the propagator for the Schrödinger equation be *K*(*t,x*|*t’,x’*).

Let the prior causal tapestry be *I* and assume that its informons embed into the lattice {(*np_T_*, *ip_L_, jp_L_*, *kp_L_*)| *n* fixed, *i*, *j*, *k* integers}. Let the corresponding bounded continuous 3-D subspace of R^4^, {(np_T_, x,y,z)| *n* fixed, *x, y, z* arbitrary real} be denoted *I* and the full unbounded subspace of R^4^ be denoted as R^4^_I_. Assume that the next generation of informons which will form the causal tapestry *I*’ embeds into the lattice {((*n* + 1)*p_T_*, *ip_L_*, *jp_L_*, *kp_L_*)| *n* fixed, *i*, *j*, *k* integers} Let us focus on the global Hilbert space interpretation and ignore the specific strategy for generating the informons. It suffices to assume that the causal manifold embedding is random with the total number of informons generated being *N*. To further simplify assume that *r* = *N*. Thus, in each causal tapestry there are *N* informons and information from all *N* prior informons will be incorporated into each nascent informon.

The global Hilbert space interpretation on the prior tapestry *I* takes the form

*Ψ_I_*(*np_T_*,*x*,*y*,*z*) = Σ_*n* in *I*_***Γ**_n_ T*(*np_T_*,*x_n_*,*y_n_*,*z_n_*)*g*(*t*,*x*,*y*,*z*)

Consider a nascent informon m which embeds in the nascent lattice at ((*n* + 1)*p_T_*,*x_m_*,*y_m_*,*z_m_*). Let its content set be *G_m_*. Then its local process strength takes the form

***Γ**_m_* = Σ*_n in I__∩Gn_ K*((*n* + 1)*p_T_*,*x_m_*,*y_m_*,*z_m_*|np_T_,x_n,_y_n_,z_n_)*p_L_^3^**Γ**_n_*

The global Hilbert space interpretation on *I*’ thus takes the form *Ψ_I’_*(*w*’) = *Ψ_I_*_’_((*n* + 1)*p_T_*,*x*,*y*,*z*) =

Σ_*m in I’*_ Σ*_n in I__∩Gn_ K*((*n* + 1)*p_T_*,*x_m_*,*y_m_*,*z_m_*|*np_T_*,*x_n_*,*y_n_*,*z_n_*)*p_L_*^3^***Γ**_n_* *T*((*n* + 1)*p_T_*,*x_m_*,*y_m_*,*z_m_*)*g*(*t*,*x*,*y*,*z*). Note that

Σ*_n in I__∩Gn_ K*((*n* + 1)*p_T_*,*x_m_*,*y_m_*,*z_m_*|*np_T_*,*x_n_*,*y_n_*,*z_n_*)*p_L_*^3^***Γ**_n_* ≈ ∫_*I*__*∩Gn*_ *K*((*n* + 1)*p_T_*,*x_m_*,*y_m_*,*z_m_*|*np_T_*,*x_n_*,*y_n_*,*z_n_*)***Γ**_n_dx*^3^

As *r* → ∞, ∫_*I*__*∩Gn*_ *K*((*n* + 1)*p_T_*,*x_m_*,*y_m_*,*z_m_*|*np_T_*,*x_n_*,*y_n_*,*z_n_*)***Γ**_n_dx*^3^ → ∫_*I*_ *K*((*n* + 1)*p_T_*,*x_m_*,*y_m_*,*z_m_*|*np_T_*,*x_n_*,*y_n_*,*z_n_*)***Γ**_n_dx*^3^

Furthermore, as *N* → ∞,

∫_*I*_*K*((*n* + 1)*p_T_*,*x_m_*,*y_m_*,*z_m_*|*np_T_*,*x_n_*,*y_n_*,*z_n_*)***Γ**_n_dx*^3^ →

∫_*R*_^4^_*I*_*K*((*n* + 1)*p_T_*,*x_m_*,*y_m_*,*z_m_*|*np_T_*,*x_n_*,*y_n_*,*z_n_*)***Γ**_n_dx*^3^ = ∫_*R*_^4^_*I*_*K*(*m*,*n*)***Γ**_n_dx*^3^

Moreover, as *r*, *N* → ∞, by Parzen’s Theorem [7], the accuracy of the global Hilbert space interpolation will extend to the entire subspace R^4^_I’_, so that.

*Ψ_I_*_’_((*n* + 1)*p_T_,x,y,z*) = Σ*_m in I’_* Σ*_n in I__∩Gn_**K*((*n* + 1)*p_T_*,*x_m_*,*y_m_*,*z_m_*|*np_T_*,*x_n_*,*y_n_*,*z_n_*)*p_L_*^3^***Γ**_n_*

*T*((n + 1)*p_T_*,*x_m_*,*y_m_*,*z_m_*)*g*(*t*,*x*,*y*,*z*) ≈

*Σ_m in I’_* ∫_*R*_^4^_*I*_*K*(*m*,*n*)*Γ_n_dx*^3^*T*((n + 1)*p_T_*,*x_m_*,*y_m_*,*z_m_*)*g*(*t*,*x*,*y*,*z*) = ∫_*R*_^4^_*I*_*K*((*n* + 1),*x*,*y*,*z*|*w*)*Γ_w_dx*^3^

In the case that *p_T_, p_L_* → 0, this simplifies to

*Ψ_I’_*(*w’*) = ∫*_R_*^4^*_I_**K*((*n* + 1),*x*,*y*,*z*|*w*)*Γ_w_dx*^3^ = ∫*_R_*^4^_*I*_*K*(*w’*|*w*)*Ψ_I_*(*w*)*dx*^3^

which is the correct form for the solution to the Schrödinger equation assuming that *Ψ_I_(w)* is the correct solution on *R^4^_I_*_._ This can be shown by an induction argument working backwards to the initial condition. Thus in the limit as the number of informons grows to cover the entire physical space and the amount of information transferred grows to become complete, and as the Planck scales diminish, the Process Algebra model will exactly reproduce the result obtained via the Schrödinger equation.

It should be clear that in a more realistic Process Algebra model, the number of informons generated by a process, the amount of information transferred from prior to nascent informons will be finite, and the Planck scale remains fixed. Thus, it can be expected that the wave function (global Hilbert space interpretation) will differ from the wave function as determined by the Schrödinger equation. Any departure from the experimentally observed value of the wave function will place a bound on the values of *N*, *r* and the minimal temporal and spatial wavelengths. Departures from the ideal wave function can occur for many reasons. If the number of informon generated per generation cycle is finite, then they will necessarily lie in a bounded spatial region. This results in a *truncation error*. Within the region the accuracy will be quite high, but it will decline outside of the region since there it will only be supported by interpolated values. If the minimal spatial and temporal wavelengths are too large then an *aliasing error* may result. The model will be unable to accurately reproduce high energy states. This is an inherent advantage since the model possesses a natural ultraviolet cutoff, but it is unclear where exactly such a cutoff lies. The choice of Planck time and length as minimal wavelengths seems natural but it may be an excessive choice. Experiments may set a larger lower bound. Too small an *r* value will result in insufficient information transfer. Together with a finite value of *N*, this will reduce the accuracy of the approximation to the kernel integral, leading to an amplitude error. Kernels which drop off rapidly with distance will suffer less from such an error. Time-jitter errors may occur if the informons are not localized exactly on a regular lattice. Finally, there may be information loss errors if the informons do not all lie on contiguous sites.

If one assumes that the minimal wavelengths are Planck scale, that *N* = *r* = 300,000, and informons are placed continguously, then the use of various estimates for the above errors [7] shows that the discrepancy between the process algebra model wave function and the corresponding Schrödinger wave function may be as small as one part in 27 [1]. The actual error is likely to be much higher but accurate estimates are challenging, especially when informons are not localized contiguously.

## Appendix C. Process Algebra

Interactions among processes are described within the Process Algebra, which possesses 9 commutative operations, and 1 non-commutative operator. The operations describe the different ways in which the timing of the generation of informons is distributed among processes, how information is shared among informons, and how compound processes are formed. These are termed couplings since the state of the processes involved in a coupling do not change as a result of the coupling. Interactions may also result in the activation or inactivation of processes, or the creation of a new process. These give rise to true interactions in which the processes involve change states or activation status, or result in new processes.

In a coupling between two processes, the processes may generate their informons simultaneously (products) or concurrently (sums). Information may be shared among the informons generated by the two processes (free) or information from each process may remain restricted to the informons generated by that process (exclusive). Each of the above has an interaction counterpart. Interactions between processes may activate an inactive process or inactivate an active process. In addition, an interaction among processes ***P*_1_**, ***P*_2_**, …, ***P***_n_ generates a new process, ***P***, which can be described in functional form as F(***P*_1_**, ***P*_2_**, …, ***P_n_***) = ***P***. If Θ(***P*_1_**, ***P*_2_**, …, ***P_n_***) describes a coupling among ***P*_1_**, ***P*_2_**, …, ***P_n_*** then the functional relation may be described using the operation of concatenation, as Θ(***P*_1_**, ***P*_2_**, …, ***P_n_***) ***P***. Sums and products are commutative and associative, and distributive. Concatenation is non-commutative and non-associative in general. There is an additional operation, “,”, which means that the two processes are independent of one another. The zero process, *O*, is the process that does nothing.

Denote the total number of informons generated by a process ***P***, that is its *N* value, by |***P***|. Then for any two processes ***P***, ***Q*** we have

|***P*** + ***Q***| = *max*{|***P***|,|***Q***|}

|***P** x **Q***| = |***P***| + |***Q***|

*This*|***PQ***| = |***Q***|
